# Transcriptomic Analysis Reveals Differentially Expressed Circular RNAs Associated with Fecundity in the Sheep Hypothalamus with Different FecB Genotypes

**DOI:** 10.3390/ani14020198

**Published:** 2024-01-07

**Authors:** Yu He, Si Chen, Xiaofei Guo, Xiaoyun He, Ran Di, Xiaosheng Zhang, Jinlong Zhang, Xiangyu Wang, Mingxing Chu

**Affiliations:** 1 State Key Laboratory of Animal Biotech Breeding, Institute of Animal Science, Chinese Academy of Agricultural Sciences (CAAS), Beijing 100193, China; heyu_22@163.com (Y.H.); chensi@westlake.edu.cn (S.C.); guoxfnongda@163.com (X.G.); hedayun@sina.cn (X.H.); diran@caas.cn (R.D.); 2 Tianjin Key Laboratory of Animal Molecular Breeding and Biotechnology, Tianjin Engineering Research Center of Animal Healthy Farming, Institute of Animal Science and Veterinary, Tianjin Academy of Agricultural Sciences, Tianjin 300381, China; zhangxs0221@126.com (X.Z.); jlzhang1010@163.com (J.Z.); 3 Jilin Provincial Key Laboratory of Grassland Farming, Jilin Province Feed Processing and Ruminant Precision Breeding Cross Regional Cooperation Technology Innovation Center, Northeast Institute of Geography and Agroecology, Chinese Academy of Sciences, Changchun 130102, China

**Keywords:** hypothalamus, FecB, circular RNAs, sheep, fecundity, follicular development

## Abstract

**Simple Summary:**

This study investigates the expression characteristics of circRNAs during follicular development in sheep with the FecB gene mutation through the use of RNA sequencing. A total of 38,979 circRNAs were identified, and differential expression was observed between different genotypes and developmental stages. Functional enrichment analysis revealed that these differentially expressed circRNAs are associated with signaling pathways such as MAPK, gap junctions, and progesterone-mediated oocyte maturation. This study also predicted a competitive endogenous RNA network, suggesting that specific circRNAs may be involved in regulating LH synthesis and secretion, thereby influencing the process of ovulation. These findings provide important insights into the role of circRNAs in follicular development and ovulation in the sheep hypothalamus and have practical implications for the improvement of sheep breeding practices.

**Abstract:**

Circular RNAs (circRNAs) are a specific type of noncoding RNA, and some have defined roles in cellular and biological processes. However, little is known about the role of circRNAs in follicular development in sheep with FecB (fecundity Booroola) mutations. Here, the expression profiles of circRNAs were investigated using RNA sequencing (RNA-seq) in the follicular phase (F) and the luteal phase (L) of FecB mutant homozygous (BB) and wild-type (WW) Small Tail Han sheep. A total of 38,979 circRNAs were identified, and 314, 343, 336, and 296 of them were differentially expressed (DE) between BB_F and BB_L, WW_F and WW_L, BB_F and WW_F, and BB_L and WW_L, respectively. The length, type, and chromosome distribution of the circRNAs and the expression characteristic between the circRNAs and their host genes in the sheep hypothalamus were ascertained. Enrichment analysis showed that the host genes of DE circRNAs in the follicular and luteal phases were annotated to MAPK, gap junctions, progesterone-mediated oocyte maturation, oocyte meiosis, and other hormone-related signaling pathways, and the different FecB genotypes were annotated to the gap junctions, circadian entrainment, MAPK, and other hormone-related signaling pathways. The competing endogenous RNA network prediction revealed that the 129 target miRNAs might be bound to 336 DE circRNAs. oar_circ_0000523 and oar_circ_0028984, which were specifically expressed during the follicular phase in the BB genotype sheep, probably acted as miRNA sponges involved in the regulation of LH synthesis and secretion. This study reveals the expression profiles and characterization of circRNAs at two phases of follicular development considering different FecB genotypes, thereby providing an improved understanding of the roles of circRNAs in the sheep hypothalamus and their involvement in follicular development and ovulation.

## 1. Introduction

Circular RNAs (circRNAs) are a novel class of functional RNA molecules that differ from linear mRNA, and they were first discovered in plant viroids more than 40 years ago [1]. These circRNAs are generated by a non-canonical splicing event named backsplicing, where a downstream 5′ splice site (splice-donor) is covalently linked to an upstream 5′ splice site (splice-acceptor) [2]. As a consequence of their covalently closed ring structure, circRNAs generally localize to the cytoplasm with high stability [3]. Although circRNAs are generally expressed at lower levels, some have 10-fold higher levels of expression than their linear counterparts [4]. Advances in high-throughput sequencing and bioinformatics techniques have helped to identify a large number of circRNAs. Furthermore, some circRNAs have been determined to have biological significance, with most of them being able to function as miRNA sponges. Cytoplasmic circAkap17b can base pair with and competitively bind to miR-7 and block the inhibitory effect of miR-7 on FSH secretion by targeting Fshb [5]. Specifically, circAkap17b has a positive regulatory role in FSH secretion in rat pituitary cells. In contrast, however, exon–intron circRNAs or intronic circRNAs function as positive regulators of their host gene transcription in a cis-acting manner by interacting with RNA pol II and U1snRNP, such as circPAIP2 and circEIF3J [6,7]. In addition, circRNAs have other functions, including ribo-circRNAs being translated into proteins or peptides through the use of the start codon of parent mRNA and binding to membrane-related ribosomes, in the regulation of alternative splicing, encoding functional proteins when they contain IRES and the m6A motif, and participating in histone modification and RNA maturation [8,9,10,11]. Taken together, these findings suggest that circRNAs deserve to be investigated and that their study may contribute to our understanding of the underlying mechanism of how the FecB gene controls complex and economically vital traits, including hypothalamic function, follicular development, and ovulation.

Small Tail Han sheep, well known as a high-fecundity breed, are widely used as female parents in crossbreeding to increase the productivity of commercial mutton sheep. Some studies have shown that the segregations of the FecB gene (Booroola fecundity) are found in Small Tail Han sheep, and the FecB gene has an additive effect on their ovulation rate and litter size that varies from the additive to the dominate B allele [12,13,14,15]. The FecB gene was initially identified in the Australian Merino sheep as a major fecundity gene, with a mutation (A746G) of the cDNA inducing a nonconservative substitution (Q249R) in the protein region of the BMPR1B gene [16]. During the folliculogenesis process, the FecB gene exerts a dramatic negative effect on FSH and LH development and progesterone production, which reduces granulosa cell production and the production of premature small antral follicles [17,18]. Meanwhile, the estrous cycle is closely associated with complex hormonal intercommunications of the hypothalamic pituitary ovarian axis [19]. Hypothalamic decapeptide GnRH can stimulate signaling cascades that confer pituitary gonadotropin hormone (FSH and LH) production, as a consequence of binding to its native high-affinity seven-transmembrane receptor (GnRHR) on the surface of the pituitary cell. Subsequently, FSH and LH exert effects on the ovaries and testes, which are conducive to steroidogenesis and gametogenesis [20]. In addition, differential GnRH pulse frequencies and amplitudes, released in a pulsatile manner from the hypothalamus, can alter the secretion patterns of FSH and LH, with decreasing frequencies leading to greater FSH release, whereas increasing frequencies lead to the preferential secretion of LH [21]. Many studies have confirmed that the development of the hypothalamus and follicular development are coordinated activities and that they are controlled by a large number of genes and noncoding RNAs [22,23,24]. Nevertheless, compared with our knowledge of hypothalamic mRNAs and miRNAs, little is known about the characterization, expression, and function of circRNAs in the hypothalamus.

In recent years, high-throughput RNA sequencing (RNA-seq) and circRNA-specific bioinformatics algorithms have identified thousands of circRNAs from a range of tissues and organisms, and have shown their tissue-specific distribution [25]. In the gilt hypothalamus, 53 circRNAs were identified to be specifically expressed in the hypothalamus, and some of them were uniquely expressed in the pre-, in-, or post-pubertal stage [26]. In sheep pituitary tissues, 12,468 circRNAs were identified in the estrus and anestrus pituitary system, and significantly enriched in hormone-related signaling pathways, neuromodulation, protein synthesis, secretion, transduction and regulation, indicating that circRNAs may be involved in the regulation of pituitary gland functions and hormone secretion [27]. In addition, 4256 circRNAs were identified in the follicular phase between monotocous and polytocous sheep ovaries [28]. Nevertheless, there have been no reports about circRNA profile in the ovine hypothalamus, with the exception of the study by Zhang et al. [24]. The study’s authors compared the expression levels of circRNAs from polytocous and monotocous FecB ++ genotype sheep hypothalamus tissues during different stages of the estrus cycle: the follicular phase and the luteal phase.

In this study, we investigated the circRNA expression profile of hypothalamus tissues from the same ewes with different FecB genotypes in the follicular phase and luteal phase, using the RNA sequencing (RNA-seq) method. We described the characteristics of these circRNAs, undertook functional enrichment analysis of the host genes of differentially expressed circRNAs, established circRNA–miRNA interactions and constructed competing endogenous RNA (ceRNA) networks to explore the role of hypothalamic circRNAs in a context in which FecB mutation affects follicular development. In addition, the linear RNA transcriptome of the same ovine hypothalamus as those used in this study was previously described by Chen et al. [22].

## 2. Materials and Methods

### 2.1. Ethics Statement

The use of the experimental sheep was endorsed by the Science Research Department of the Institute of Animal Sciences, Chinese Academy of Agriculture Sciences (IAS-CAAS). The ethical approval granted followed the guidelines of the Animal Ethics Committee of the IAS-CAAS (No. IAS 2019-49).

### 2.2. Animals and Tissue Samples

Based on TaqMan genotyping of the FecB gene in sheep [29], six wild-type (WW) and six *FecB* mutant homozygous (BB) non-pregnant ewes were randomly selected from a Small Tail Han sheep nucleus herd (Yuncheng, China), which had free access to food and water under natural conditions (lighting and temperature). The selected sheep were 3 years old and of similar weight, and showed significant differences in terms of ovulation rate and litter size (Table 1). Following these steps, we divided the selected sheep into follicular and luteal phase groups. First, the sheep were treated for estrus synchronization, including the insertion of vaginal sponges (progesterone 300 mg, InterAg Co., Ltd., Hamilton, New Zealand), and injection with vitamin A and D. Second, the vaginal sponges were withdrawn after 12 days, with the removal time being set as 0 h. On the basis of previous ovine reproductive trait analyses [13,14], six ewes (three BB genotype and three WW genotype) were euthanized at 45 h after evacuation of the vaginal sponges (follicular phase), and six ewes (three BB genotype and three WW genotype) were euthanized at 216 h after evacuation of the vaginal sponges (luteal phase). Hypothalamic tissues were dissected and frozen in liquid nitrogen immediately after euthanasia.

### 2.3. RNA Isolation, Library Construction and Sequencing Analysis

Total RNA from the hypothalamic tissues was isolated using TRIzol reagent (Thermo Fisher Scientific, Waltham, MA, USA). A Nano Photometer^®^ spectrophotometer (IMPLEN, Westlake Village, CA, USA) and Qubit^®^ RNA Assay kits (Thermo Fisher Scientific) were used to characterize the purity and concentration of the total RNA extracted, respectively. The RNA integrity number (RIN) value of all samples was determined to select high-quality RNA samples (RIN > 7), and ribosomal RNA (rRNA) was depleted using Ribo-Zero™ GoldKits (Epicentre, Madison, WI, USA). The remaining hypothalamus RNA (3 μg) was taken as a starting amount to construct sequencing libraries, using NEB Next Ultra Directional RNA Library Prep Kit for Illumina (NEB, Ipswich, MA, USA) in accordance with the manufacturer’s recommendations. Then, the pooled libraries were subjected to paired-end sequencing by Hiseq X (Illumina, San Diego, CA, USA). All sequencing data were outsourced to Annoroad Gene Technology Co., Ltd. (Beijing, China).

Illumina high-throughput sequencing was processed by CASAVA (v.1.8.0) to generate raw sequenced reads on base calling. Based on in-house Perl scripts of Annoroad Gene Technology Co., Ltd. (Beijing, China), clean data were obtained by removing raw reads with adapter contamination, poly-N > 5%, low quality (quality scores < Q30), or matched to rRNA sequences [30]. Q30 means that the different base sequencing error rate is 0.1%. The obtained clean reads were then mapped to the ovine genome Oar_v4.0 (https://www.ncbi.nlm.nih.gov/assembly/GCF_000298735.2, accessed on 9 October 2023) by the BWA-MEM algorithm of BWA (v.0.7.15) [31], which was recommended by the circRNA identifier (CIRI, v.1.2) program.

### 2.4. Identification of Potential circRNA

CIRI (v.1.2) was employed to detect and identify circRNA candidates, which scanned sequence alignment map (SAM) files twice and collected sufficient information [32]. During the first scanning step, for the SAM files produced by the BWA program, CIRI detected balanced junction reads with paired chiastic clipping (PCC) signals in accordance with the CIGAR values, which were identified as potential circRNAs. Preliminary filtering was performed by paired-end mapping (PEM) and GT-AG splicing signals, in which the PEM results were used to filter false positive PCC signals, and GT-AG splicing signals were utilized for the detection of circRNAs. Subsequently, CIRI scanned the SAM files again using dynamic programming, with it discovering additional junction reads, and implemented a further filter to prevent false predictions due to the similarity of repetitive sequences and homologous genes.

### 2.5. Differential Expression Analysis of circRNA

Based on the identification of CIRI and annotation of the ovine reference genome Oar_v4.0, a statistical analysis was conducted on the types, length, and chromosomal distribution of the circRNA candidates. The expression levels of annotated circRNAs were normalized using the spliced reads per billion mapping (SRPBM) approach, which allowed for quantitative comparisons between backsplices [33]. Due to there being three biological replicates in each group, the differentially expressed (DE) circRNAs were screened by the DEseq2 package (v.1.28.1) with the parameters of |Fold Change| > 1.5 and the *p*-value < 0.05 [34], which were significantly differentially expressed in BB_F vs. BB_L, WW_F vs. WW_L, BB_F vs. WW_F, and BB_L vs. WW_L, respectively. According to the log2(SRPBM) value of each DE circRNA, a systematic clustering analysis was performed by pheatmap (v.1.0.2) to directly reflect circRNA expression levels under different conditions [35]. The systematic clustering analysis involved the Euclidean distance and Pearson’s correlation methods. It is generally suggested that function-related genes or RNAs have similar expression patterns under the same conditions.

### 2.6. Functional Annotation of Target Genes with DE circRNA

To clarify the potential roles of the circRNA’s target genes, Gene Ontology (GO, http://www.geneontology.org, accessed on 9 October 2023) and Kyoto Encyclopedia of Genes and Genomes (KEGG) statistical enrichment analysis were carried out using the clusterProfiler package (v3.16.0). GO is a collection of controlled vocabularies for gene functions organized into three categories: molecular function (MF), biological process (BP), and cellular component (CC). Among them, BP is defined as a biological objective in which the gene or gene product is involved, MF refers to the biochemical activity of a gene product, and CC represents the cellular place where a gene product is active [36]. Pathway analysis by KEGG (http://www.kegg.jp, accessed on 9 October 2023) shows that gene catalogs from fully sequenced genomes are related to system functions [37]. That is, it can identify that host genes are related to major signal transduction pathways and biochemical metabolic pathways through use of the KEGG database. The method for correcting the *p*-value (q-value) was the use of the false discovery rate (FDR). GO terms and KEGG pathways (q-value) were defined as being significantly enriched.

### 2.7. Binding Site Prediction of DE circRNA

CircRNA acting as a miRNA sponge can inhibit the negative role of miRNA on target mRNAs at specific sites. Since circRNA sequences have multiple miRNA binding sites, we predicted the relationship of circRNA binding to miRNA using miRanda software (v.3.3a, http://www.microrna.org, accessed on 9 October 2023), whose algorithm improves sequence complementarity using position-specific rules and strictly depends on interspecies conservation [38]. The circRNA-miRNA-mRNA interaction networks were visualized with Cytoscape (v3.8.0)

### 2.8. Validation of circRNAs

Total RNAs from ovine hypothalamic tissues were reverse-transcribed to cDNA using a PrimeScript™ RT Reagent Kit with gDNA Eraser (Takara, Beijing, China). To confirm the presence of circRNAs, we performed reverse transcription PCR (RT-PCR) using specific primers for several DE circRNAs, which were designed using Primer Premier 6.0 and synthesized by Sangon Biotech (Beijing, China; Table S1). RT-qPCR was performed under the following conditions: 95 °C for 5 min, followed by 40 cycles of amplification at 95 °C for 30 s and annealing at 60 °C for 30 s. The RT-PCR products were amplified and Sanger sequenced. See Figure S1 in the Supplementary Information for the peak of the Sanger sequencing results. Subsequently, several DE circRNAs were subjected to the real-time quantitative PCR (RT-qPCR) to support the reliability of the RNA-seq results. The primers utilized for the RT-qPCR were the same as those for the RT-PCR. The RT-qPCR was performed in triplicate by using TB Green^®^ Premix Ex Taq™ II (Takara, Beijing, China) on a Roche Light Cycler^®^ 480 II system (Roche Applied Science, Mannheim, Germany). The sheep actin beta (ACTB) gene was used as a control [39]. The relative expression levels of DE circRNAs were analyzed through the use of the 2^−ΔΔCt^ method [40]. Between the RT-qPCR results and the FPKM values from RNA-seq, the correlation was implemented using the Pearson correlation method of R (v.4.0.2).

## 3. Results

### 3.1. Overview of Sequencing Data in Small Tail Han Sheep Hypothalamic Tissue

All of the raw reads presented in this study have been available in online repositories with the accession number PRJNA672275. On average, 1,442,109,616 raw reads were obtained from the 12 ovine hypothalamic tissues. The accuracy of base recognition (Q30) from each sample was over 90.89%. After quality control, at least 99.97% of clean reads were mapped to the ovine reference genome assembly in each library, which involved the unique mapped reads reaching 75.48% and multiple mapped reads reaching less than 17.35% (Table S2).

### 3.2. Characterization of circRNAs in Small Tail Han Sheep Hypothalamic Tissue

**Figure 1:**
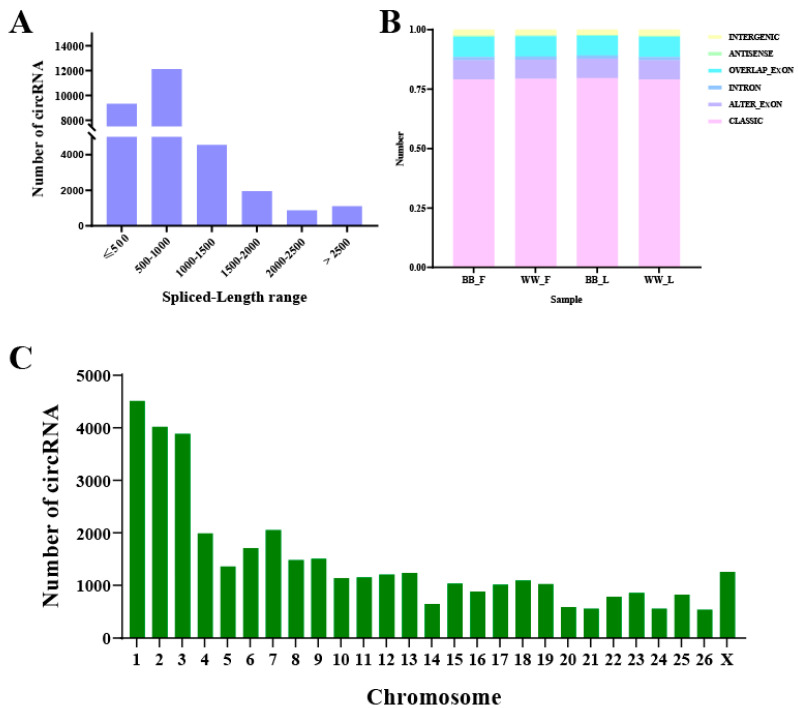
General characteristics of the circRNAs in the sheep hypothalamus. (**A**) The length distribution of the circRNAs. (**B**) The genomic regions distribution of the circRNAs. (**C**) The chromosomal location of the circRNAs.

After mapping, 38,979 circRNAs were annotated to the ovine reference genome. The spliced length distribution of these circRNAs ranged from tens to thousands of bases but was primarily concentrated below 1000 bases (Figure 1A). Of the six circRNA types identified, the splicing exons were the most common sequences identified, accounting for 78.88%, 79.59%, 79.19%, and 79.05% of the circRNAs in the BB_F, BB_L, WW_F, and WW_L, respectively. Intronic was the least common type (1.32%, 1.41%, 1.39%, and 1.36%) of all of the circRNAs (Figure 1B). These circRNAs were widely distributed on ovine chromosome 1 to X, and the greatest number (approximately 31.88%) were located on chromosomes 1, 2, and 3 (Figure 1C). In addition, novel circRNAs were renamed after the parental genes (Table S3).

### 3.3. The Profiling of DE circRNAs in Small Tail Han Sheep Hypothalamic Tissues

**Figure 2:**
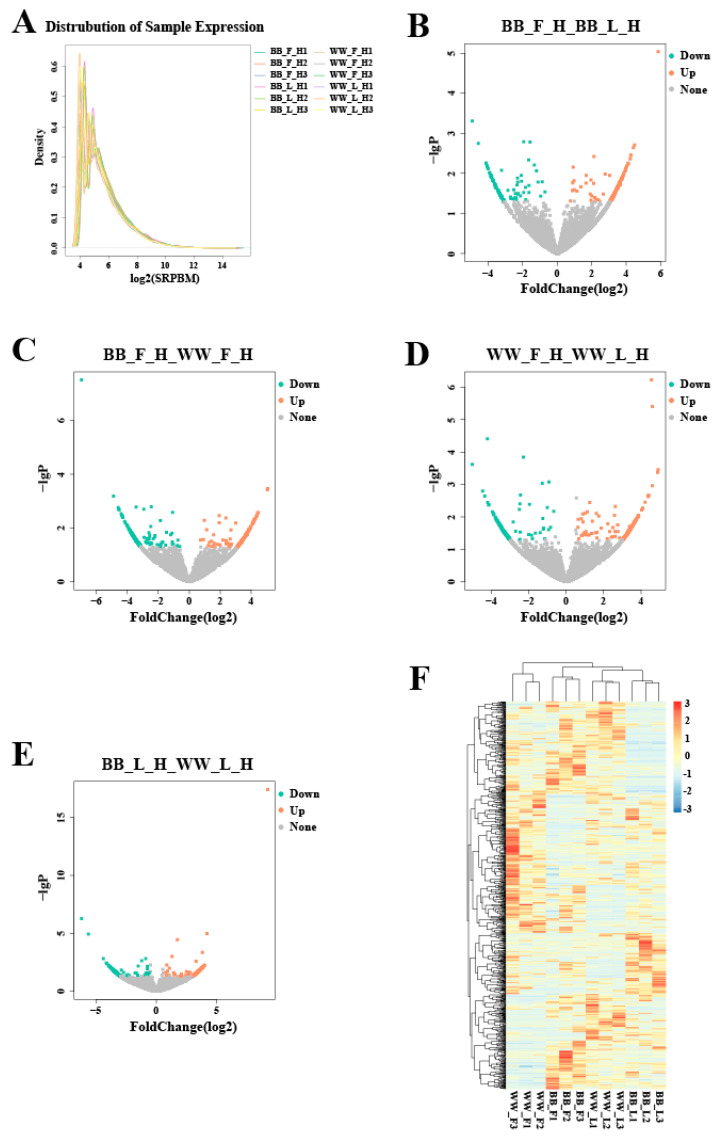
Screening and clustering analysis of DE circRNAs. (**A**) The expression level distribution of DE circRNAs in the follicular and luteal with different FecB genotypes. The expression was normalized with SRPBM. (**B**–**E**) Volcano plots showing the upregulated and downregulated DE circRNAs in BB_F vs. BB_L (**B**), in BB_F vs. WW_F (**C**), in WW_F vs. WW_L (**D**), and in BB_L vs. WW_L (**E**). (**F**) Hierarchical clusters of DE circRNAs.

The expression levels of circRNAs in BB_F, BB_L, WW_F, and WW_L were calculated, and the expression levels were normalized through the use of SRPBM (Figure 2A). Under the normalized expression with |Fold Change| > 1.5 and *p*-value < 0.05, 314 DE circRNAs (171 upregulated and 143 downregulated) were screened between BB_F and BB_L, involving oar_circ_0018153, oar_circ_0028984, oar_circ_0022103, oar_circ_0022016, oar_circ_0030238, and oar_circ_0005502, which were produced by the genes *SOS2*, *PIK3R1*, *KCNQ5*, *RIMS2*, *RAPGEF2*, and *RBMS1*, respectively (Figure 2B, Table S4). In BB_F and WW_F, 336 DE circRNAs (149 upregulated and 187 downregulated) were identified, among which oar_circ_0000523, oar_circ_0005502, oar_circ_0018158, oar_circ_0032202, oar_circ_0001768, and oar_circ_0006372 originated from the genes *USP25*, *RBMS1*, *SOS2*, *ITPR1*, *PIK3CA*, and *BMPR2*, respectively (Figure 2C, Table S4). Between WW_F and WW_L, 343 DE circRNAs (194 upregulated and 149 downregulated) were screened, including oar_circ_0008497, oar_circ_0003861, oar_circ_0007731, oar_circ_0013269, oar_circ_0019258, oar_circ_0031930, and oar_circ_0015097, which were generated by the genes *TEK*, *KIAA1107*, *GNAQ*, *GNAI1*, *TGFB3*, *IGF1R*, and *CDC23*, respectively (Figure 2D, Table S4). In BB_L and WW_L, 296 DE circRNAs (129 upregulated and 167 downregulated) were identified, among which oar_circ_0001872, oar_circ_0010231, oar_circ_0030918, oar_circ_0012515, oar_circ_0015496, oar_circ_0037084, and oar_circ_0007733 were produced by the genes *PHC3*, *DENND58*, *CHD2*, *BRAF*, *RASA1*, *GNG4*, and *GNAQ*, respectively (Figure 2E, Table S4). To analyze the cluster model of circRNA differential expression in each of the four pairwise comparisons, 1049 DE circRNAs were clustered through the use of K-means clustering analysis (Figure 2F).

### 3.4. Enrichment Analysis of DE circRNAs

**Figure 3:**
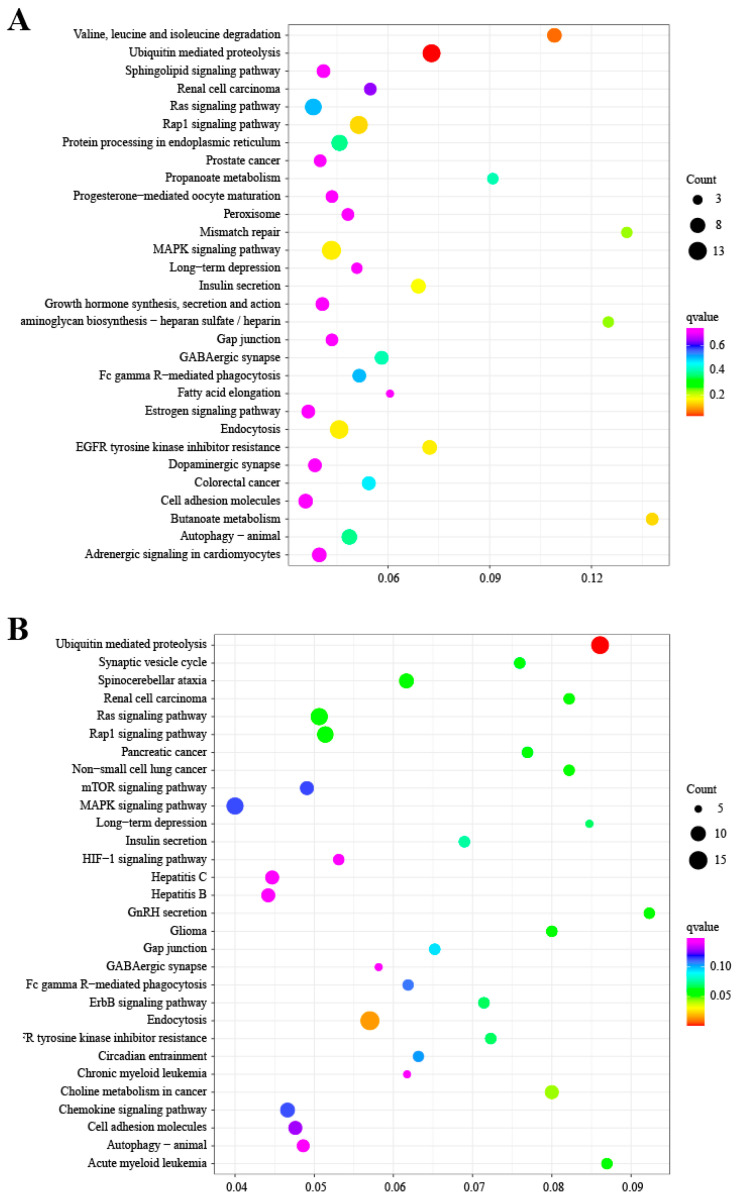
Enrichment analysis of the host genes of the DE circRNAs. (**A**) KEGG enrichment pathways the host genes of DE circRNAs in the follicular phase versus the luteal phase. (**B**) KEGG enrichment pathways the host genes of DE circRNAs in the BB (FecB-mutant homozygous) versus WW (wild type) genotypes. The X-axis is Rich_ Ratio, the Y-axis is pathway.

To further explore the potential functions of DE circRNAs between the follicular phase and the luteal phase, GO and KEGG enrichment analysis of the host genes of the DE circRNAs was performed. Based on three categories of GO, the most enriched biological process (BP) terms were cellular process, single-organism process, biological regulation, and metabolic process; the most enriched molecular function (MF) terms were binding, catalytic, molecular function regulator, and transporter; and the most enriched cellular component (CC) terms were cell part, organelle, membrane, and membrane part (Table S5). Comparison of KEGG pathway databases and host genes revealed that the host genes were annotated into 242 pathways, including the MAPK signaling pathway, insulin secretion, the Ras signaling pathway, growth hormone synthesis, secretion and action, gap junctions, progesterone-mediated oocyte maturation, the estrogen signaling pathway, oocyte meiosis, the TGF-beta signaling pathway, and other pathways (Figure 3A, Table S5).

Between genotype BB and genotype WW, the GO results showed that the most enriched BP terms were cellular process, biological regulation, single-organism process, cellular component organization or biogenesis, and metabolic process; the most enriched MF terms were binding, catalytic, molecular function regulator, and molecular transducer; and the most enriched CC terms were cell part, organelle, organelle part, and membrane part (Table S5). According to the results of the KEGG enrichment analysis, the host genes were annotated into 251 pathways, including GnRH secretion, the Ras signaling pathway, the ErbB signaling pathway, gap junctions, circadian entrainment, the MAPK signaling pathway, the GABAergic synapse, the GnRH signaling pathway, the oxytocin signaling pathway, and other pathways (Figure 3B, Table S5).

### 3.5. Bioinformatic Analysis of circRNA-miRNA Networks

**Figure 4:**
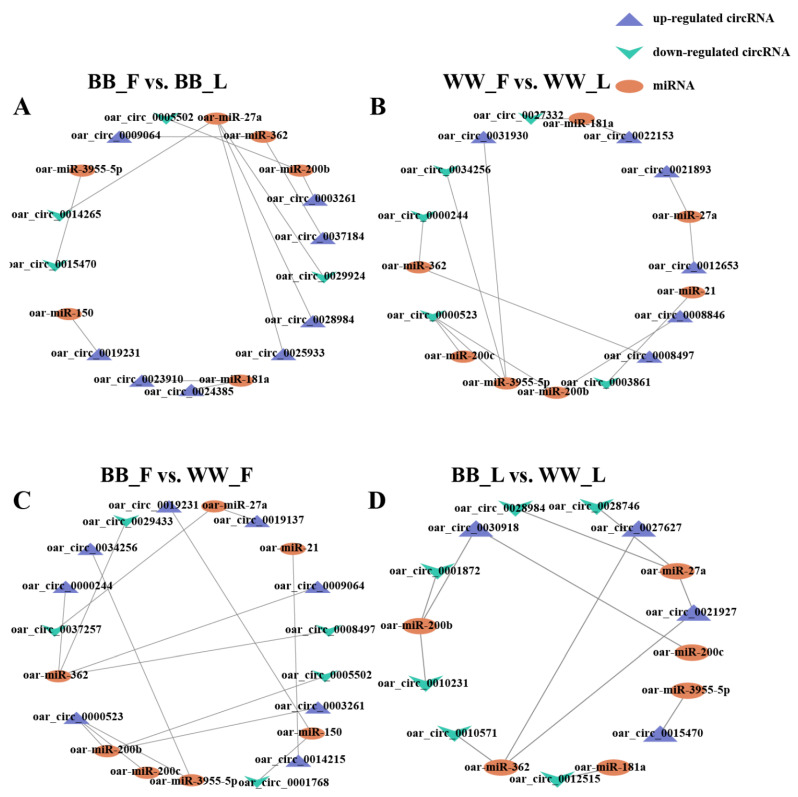
Interactions of the DE circRNAs with miRNAs form a network. (**A**) Comparison of BB_F vs. BB_L. Green and blue colors denote upregulated and downregulated DE circRNAs, respectively. Triangle and ellipse denote circRNAs and miRNAs, respectively. (**B**) Comparison of WW_F vs. WW_L. (**C**) Comparison of BB_F vs. WW_F. (**D**) In the comparison BB_L vs. WW_L. F, follicular phase; L, luteal phase; BB, FecB-mutant homozygous genotype; WW, FecB wild-type genotype.

To further explore the function of circRNAs in the hypothalamus regulatory network of Small Tail Han sheep, miRanda software (v.3.3a) was used to predict circRNA–miRNA binding. The results showed that 129 miRNAs might be bound to 336 DE circRNAs (Table S6). To increase the visuality of networks, 12, 11, 13, and 10 DE circRNAs in the BF vs.BL, WF vs. WL, BF vs. WF, and BL vs. WL groups, respectively, and their corresponding target miRNAs with higher scores and higher absolute value of total energy were screened to construct circRNA–miRNA networks (Figure 4). Some miRNAs that have been previously associated with ovine follicle development and hormone synthesis, including miR-21, miR-27a, miR-150, miR-181a, miR-200b, miR-200c, miR-362, and miR-3955-5p, were found [41,42,43].

### 3.6. Identification of Circular RNAs in Sheep Hypothalamic Tissue

**Figure 5:**
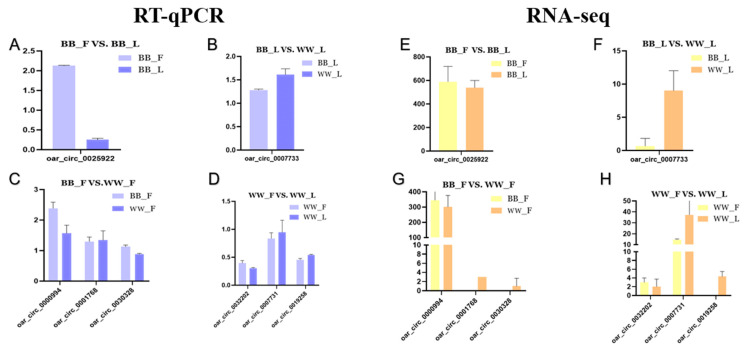
Validation of the RNA sequencing (RNA-seq) data by real-time quantitative PCR (RT-qPCR). The RT-qPCR data are presented as relative quantity. Selected genes from BF and BL (**A**,**E**) and from WF and WL (**D**,**H**) were validated by RT-qPCR and RNA-seq, respectively. BF and WF (**C**,**G**) and from BL and WL (**B**,**F**) were validated by RT-qPCR and RNA-seq, respectively. BF, FecB-mutant homozygous genotype ewes in the phase; BL, FecB-mutant homozygous genotype ewes in the luteal phase; WF, FecB wild-type genotype ewes in the follicular phase; WL, FecB wild-type genotype ewes in the luteal phase.

To validate the RNA-seq data, randomly selected circRNAs were examined through the use of RT-qPCR, and the relative gene expression was calculated using the 2^−ΔΔCt^ method (Figure 5). The obtained results indicated that the data regarding the circRNAs’ expression levels were reliable. Meanwhile, the results of the Pearson’s correlation analysis of all genes showed that there was a strong positive correlation between the RT-qPCR and RNA-seq data (cor > 0.93, *p* < 0.05).

## 4. Discussion

In the ovine estrous cycle, hypothalamic hormones and factors regulate the secretion of various downstream glandular hormones that participate in the reproductive process. During follicle development in ewes, a single autosomal gene (FecB) attenuates the positive effect of BMP on granulosa cell mitosis, enabling higher FSH sensitivity and enhanced expression of the LH receptor, which promotes smaller follicles’ maturation and ovulation [17]. Clarke reported a marked increase in GnRH secretion at the onset of the LH surge (Smith,2011). RNA-seq can accurately identify and characterize low expression levels of circRNA. In recent years, multiple circRNAs have been annotated to the reproductive process based on RNA-seq. One of the weaknesses in analyzing RNA-seq data is measurement noise. Due to its high accuracy and sensitivity, RT-qPCR has been used in the validation of circRNA expressions, with them being identified by RNA-seq in a variety of species [42,43,44,45].

In our study, the results based on CIRI, in that the majority (76.10%) of circRNAs originated from protein-coding exons, is consistent with findings found in sheep, goats, mice, and humans [46,47,48,49], which indicates the circRNA specificity and preference of some genes [50]. In comparison with circRNAs’ presence in other ovine tissues, the total number of circRNAs identified (38,979) was more than has been described for pituitary and ovarian tissues, with 12,468 and 4256 circRNA being identified, respectively [27,28], which reflects circRNAs’ tissue-specific expression patterns and the specific character of the different tissues. In addition, previous studies have reported that circRNAs from humans and pigs have different tissue-specific expression patterns [51,52]. The length of most circRNAs was found to be less than 1 kb, which is consistent with other circRNA transcriptome analyses on the ovine hypothalamus and mammary gland [53]. Furthermore, the distribution of most circRNAs covered chromosomes 1 to 3, as these chromosomes comprise 28.61% of the total length of the ovine genome (Oar_v4.0).

This study collected follicular phase samples from ewes within 45 h after the insertion and removal of vaginal sponges, covering two estrous cycles. We identified 318 and 345 differentially expressed circular RNAs (circRNAs) between the follicular and luteal phases in ewes with BB and WW genotypes, respectively. Enrichment analysis revealed that parental genes were involved in multiple signaling pathways related to oocyte meiosis, hormone-mediated oocyte maturation, estrogen signaling, gap junctions, circadian rhythm disruption, and insulin secretion. Importantly, in the WW genotype, GNAQ directly regulated GnRH expression and secretion, decreased downstream ovarian gene expression, and regulated the ewe’s estrous cycle [54]. KIAA1107, CDC23, IGF1R, and GNAI1 were found to be required for normal synaptic transmission, oocyte meiosis, folliculogenesis, and ovulation, respectively [55,56,57]. In the BB genotype, we found that the host genes of oar_circ_0028984, oar_circ_0005502, oar_circ_0022103, oar_circ_0030238, and oar_circ_0022016 were PIK3R1, RBMS1, KCNQ5, RAPGEF2, and RIMS2, respectively. Enrichment analysis showed that PIK3R1 was associated with prolactin, ErbB, estrogen, and cholinergic synaptic signaling pathways [58]. RBMS1 played an important role in regulating oocyte maturation and the estrous cycle by positively increasing CYP19A1 expression and estradiol secretion. KCNQ5, RIMS2, and RAPGEF2 established connections between neurons and endocrine cells, such as estrogen treatment increasing KCNQ5 expression, which is crucial for regulating neural excitability [59,60,61]. In the wild-type ewes, parental genes primarily influence the expression and secretion of hormones involved in oocyte meiosis, follicle development, and ovulation. Meanwhile, the FecB gene mutation in parental sheep is associated with neural–endocrine cell connections, leading to the increased expression of hormone-related and neural excitatory genes.

Our previous studies have shown that as the copy number of the B allele increases (*p* < 0.01), ovulation rates and E2 and FSH concentrations increase, while the diameter of pre-ovulation follicles decreases [13,14]. However, the specific mechanism of the FecB gene’s effect on ovulation remains unclear. In further exploring this, we selected two genotypes of sheep and found 338 differentially expressed circRNAs in the follicular phase (BB_F vs. WW_F) and 299 circRNAs in the luteal phase (BB_L vs. WW_L). These numbers are similar to a previous study on polytocous and monotocous sheep, which identified 333 and 340 circRNAs in the follicular and luteal phases, respectively [24]. In the FecB-mediated follicular phase, genes such as SOS2, ITPR1, PIK3CA, USP25, RBMS1, and BMPR2 produced specific circRNAs and were involved in reproduction-related signaling pathways. [62,63,64]. Meanwhile, in the FecB-mediated luteal phase, eight differentially expressed circRNAs originated from genes related to the regulation of gene expression, cellular proliferation and differentiation, and transmembrane signal transduction [65,66,67,68,69,70,71]. These findings shed light on the complex mechanisms through which the FecB gene influences the follicular and luteal phases in sheep.

Previous studies have reported that some circRNAs are expressed at ten-fold higher levels than canonical linear mRNA, although circRNAs frequently accumulate at low abundance [72]. In comparison with the elevated expression levels of mRNAs in the hypothalamus transcriptome analysis of Chen et al. [22], the expressed levels of circRNAs were comparatively high. For example, the same study reported that the FPKM values of RAPGEF2 and SOS2 were 14 and 21, respectively, in the follicular phase of the BB genotype sheep. However, the circRNAs were expressed at 11- and 61-fold higher levels than these gene transcripts, respectively. This is consistent with the findings of Hao et al. in sheep, where it was also observed that some circRNAs were expressed at higher levels than their parent genes. Meanwhile, it was suggested that for the cis-acting of circRNAs on their host genes, individual circRNAs were not necessarily at high abundance for the effect to occur [7]. Taken together, this would suggest that these DE circRNAs and their host genes might play similar roles in the follicular and luteal phases under different FecB genotypes.

In contrast, for circ_0000523 in this study, there was no clear correlation (r = 0.16) in the expression of three pooled samples with its host gene USP25 in a previous study during the follicular phase of the WW genotypes [73]. The failure to find such a relationship may be due to circRNA acting as an miRNA sponge and not necessarily owing to activity associated with the host gene. Similarly, Wilusz implied that there is often no obvious relationship between the expression levels of circRNAs and their parent genes [50]. Notably, the type of these circRNAs is predominantly exonic circRNA. Exonic circRNAs primarily localize to the cytoplasm and function as miRNA sponges [73]. MiRNAs, a type of small noncoding RNAs, post-transcriptionally regulate gene expression by repressing translation or degrading target mRNAs [74]. Increasingly evidence implies that circRNAs thereby relieve or inhibit the repression of target mRNAs as miRNA sponges, which is a general phenomenon [75]. Furthermore, circRNAs seemed to interact with a variety of miRNAs, whereas other circRNAs only appeared to sponge a single miRNA. In our study, circ_0019230 could potentially interact with 49 target miRNAs, while circ_0028984, circ_0031930, oar_circ_0008497, circ_0001768, and oar_circ_0012515 seemed to only sponge a single miRNA, which is consistent with a previous study on ovine pituitary cells, with the study’s authors reporting that circ_0011850 seemed to sponge 15 miRNAs, while circ_0007177, circ_0008176, circ_0009594, and circ_0009624 only appeared to interact with miR-107 [27].

It is worth mentioning that circRNAs derived from ovarian cells in extracellular vesicles play a crucial role in regulating gene expression, affecting ovarian cell growth, function, and the development of ovarian diseases [76,77]. Future research will provide new insights into diagnosis and treatment methods.

In this study, we predicted the target miRNAs of DE circRNAs through the use of miRanda software (v.3.3a). We discovered a range of target miRNAs that are closely related to follicular development, ovulation, and the regulation of reproductive hormone synthesis. For instance, circ_0003861 was found to interact with miR-21, which is known to enhance the growth of preovulatory follicles and protect granulosa cells against apoptosis [78]. Additionally, circ_0031930 exhibited an interaction with miR-3955-5p, a sheep-specific miRNA that could potentially play a role in reproductive processes [63]. A disruption in BMP signaling may negatively affect granulosa cell proliferation, oocyte maturation, ovulation, steroidogenesis, and oocyte–somatic cell communication, which could lead to reproductive abnormalities and infertility [79]. miR-3955-5p has only been identified in sheep [27] and has not been annotated in the miRbase database of other species, indicating that miR-3955-5p might be an Ovis aries-specific transcript [80]. Furthermore, we observed that, under the influence of the FecB gene, several circRNAs were predicted to sponge miRNAs, such as miR-200b, miR-27a, and miR-150, which may impact follicular–luteal transition and the function of granulosa cells [81,82,83]. Taken together, circRNA–miRNA interactions will require in-depth research as candidates in follow-up functional studies.

## 5. Conclusions

We identified the characterization and expression profiles of circRNAs from hypothalamus transcriptome with the FecB mutation in the follicular–luteal transition. Functional enrichment analysis showed that some DE circRNAs in the follicular and luteal phases played roles in oocyte maturation and hormone-related signaling pathways. The analysis of the ceRNA network suggests that oar_circ_0000523 and oar_circ_0028984 are potentially involved in the regulation of LH synthesis and secretion, thereby influencing the process of ovulation. The circRNA data in the hypothalamus of sheep obtained in this study should provide references for sheep prolificacy and helpful sheep breeding.

## Figures and Tables

**Table 1:** Reproduction trait and sample information of the ewes with different FecB genotypes.

Period	Genotype	Age (Years)	Body Weight (kg)	Litter Size	Ovulation Number	Follicles Number	Corpus Luteum Number
Luteal phase	BB	2.66 ± 0.57	64.00 ± 11.13	2.33 ± 0.57	2.66 ± 1.15	-	2.66 ± 0.57
	WW	1.83 ± 0.76	73.00 ± 11.78	1.00	1.33 ± 0.57	-	1.00
follicular phase	BB	2.50 ± 0.86	67.33 ± 5.03	2.33 ± 0.57	3.33 ± 1.15	4.33 ± 2.51	-
	WW	1.50 ± 0.50	71.00 ± 3.00	1.00	1.00	1.00	-

The data were represented as mean ± standard error.

## Data Availability

The datasets presented in this study are available in online repositories (https://www.ncbi.nlm.nih.gov/sra/PRJNA672275, accessed on 9 October 2023).

## Supplementary Materials

The following supporting information can be downloaded at: https://www.mdpi.com/article/10.3390/ani14020198/s1, Figure S1: Sanger sequencing results of the RT-PCR products of circRNAs; Table S1: Primer sequence and product size of circRNAs for RT-qPCR; Table S2: Alignment of statistical results of reads; Table S3: All circRNAs identified in hypothalamus tissues; Table S4: DE circRNAs identified in hypothalamus tissues; Table S5: Enrichment analysis of DE circRNAs; Table S6: Bioinformatic analysis of circRNA-miRNA networks.
