# Supplementary material for: Transcriptomic Analysis Reveals Differentially Expressed Circular RNAs Associated with Fecundity in the Sheep Hypothalamus with Different FecB Genotypes
Source: Animals (Basel). 2024 Jan 7;14(2):198. doi: 10.3390/ani14020198 (PMC10812736; doi:10.3390/ani14020198)

Figure S1 Sanger sequencing results of the RT-PCR products of circRNAs

Sanger sequencing results of the RT-PCR product of oar_circ_0025922

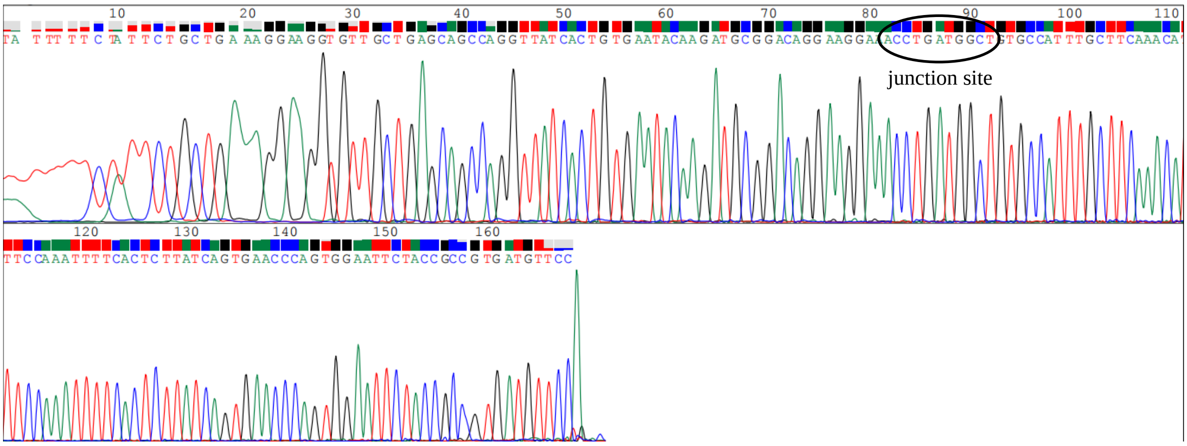

Sanger sequencing results of the RT-PCR product of oar_circ_0001768

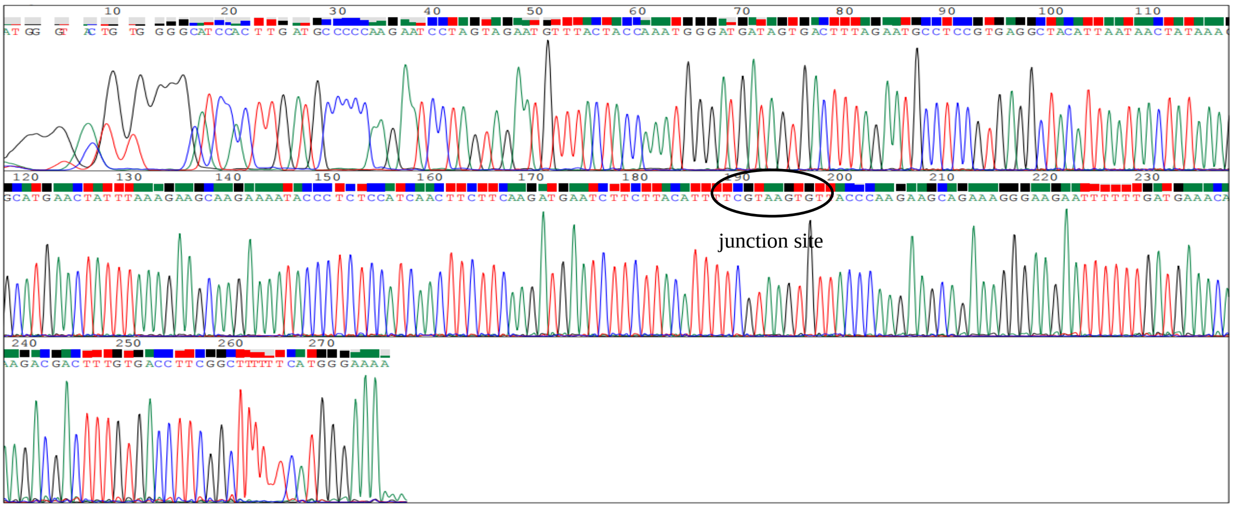

Sanger sequencing results of the RT-PCR product of oar_circ_0000994

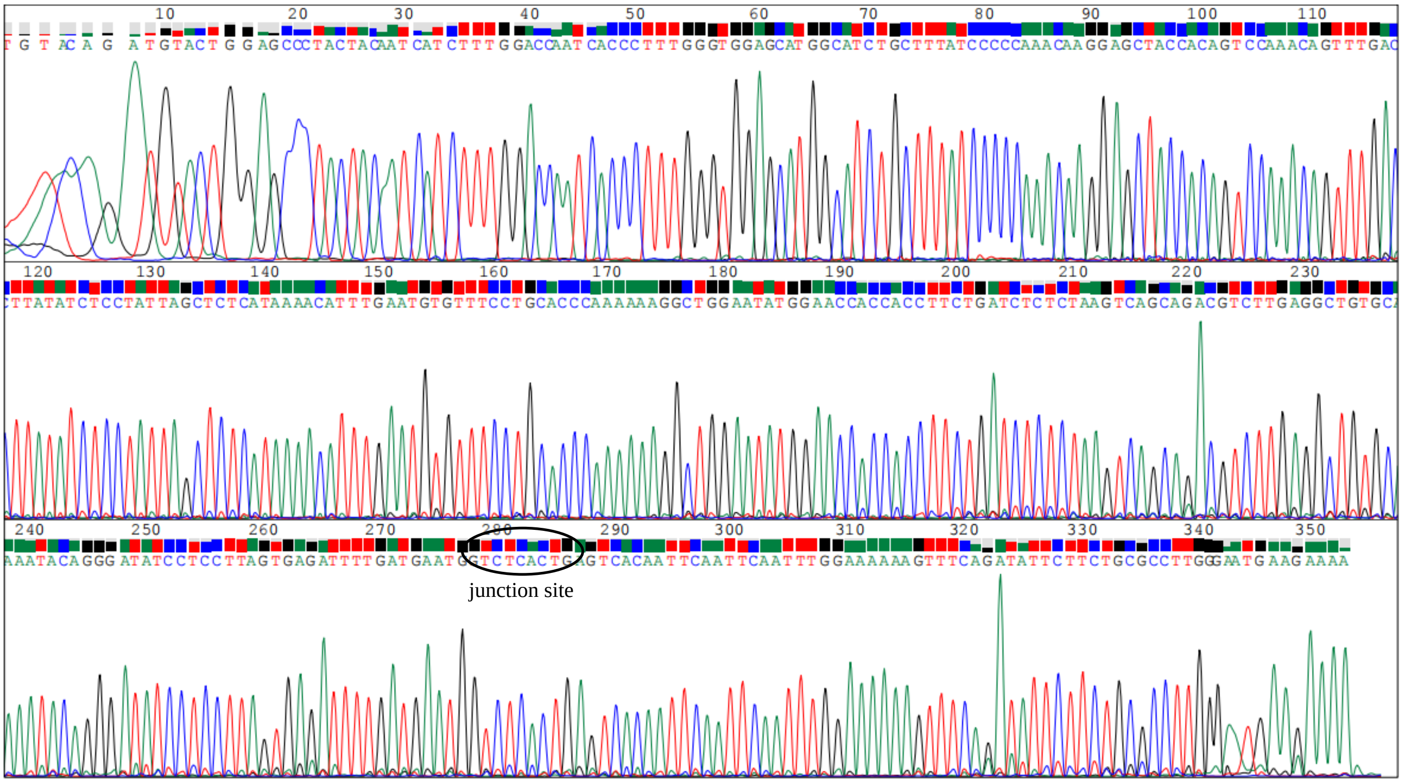

Sanger sequencing results of the RT-PCR product of oar_circ_0032202

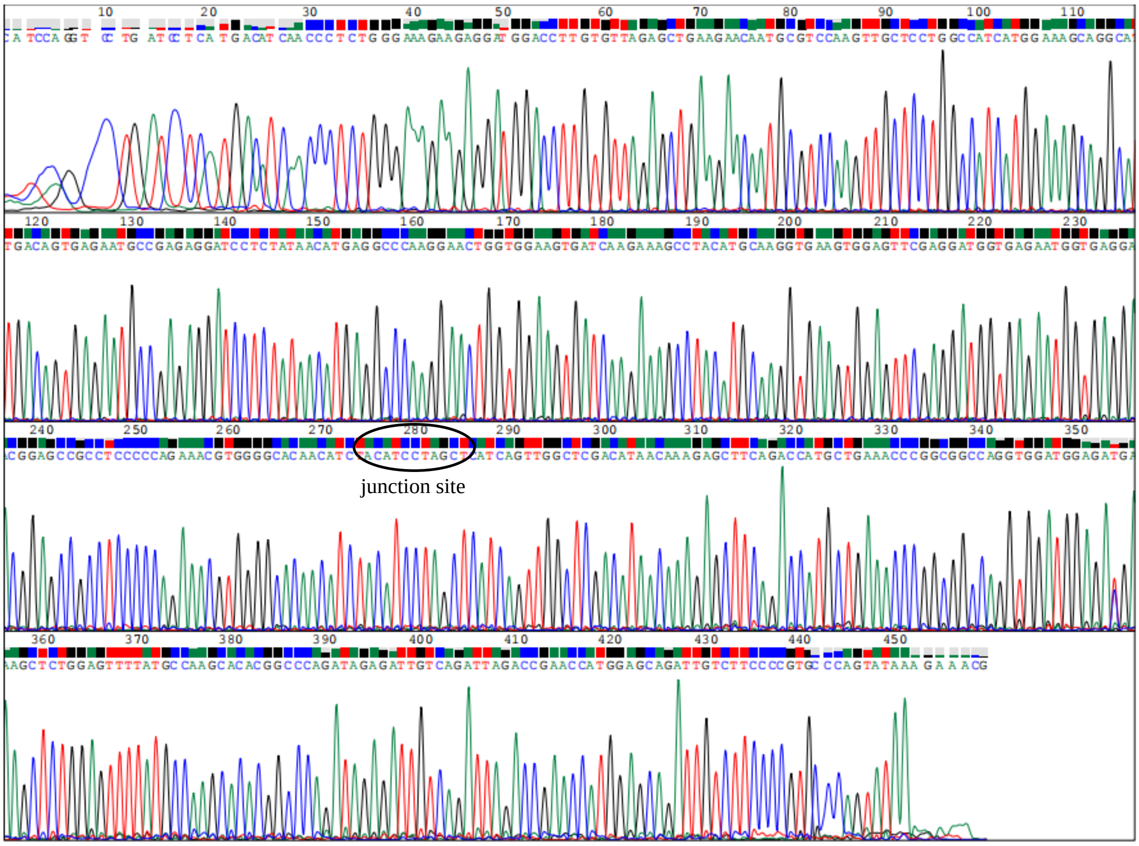

Sanger sequencing results of the RT-PCR product of oar_circ_0030328

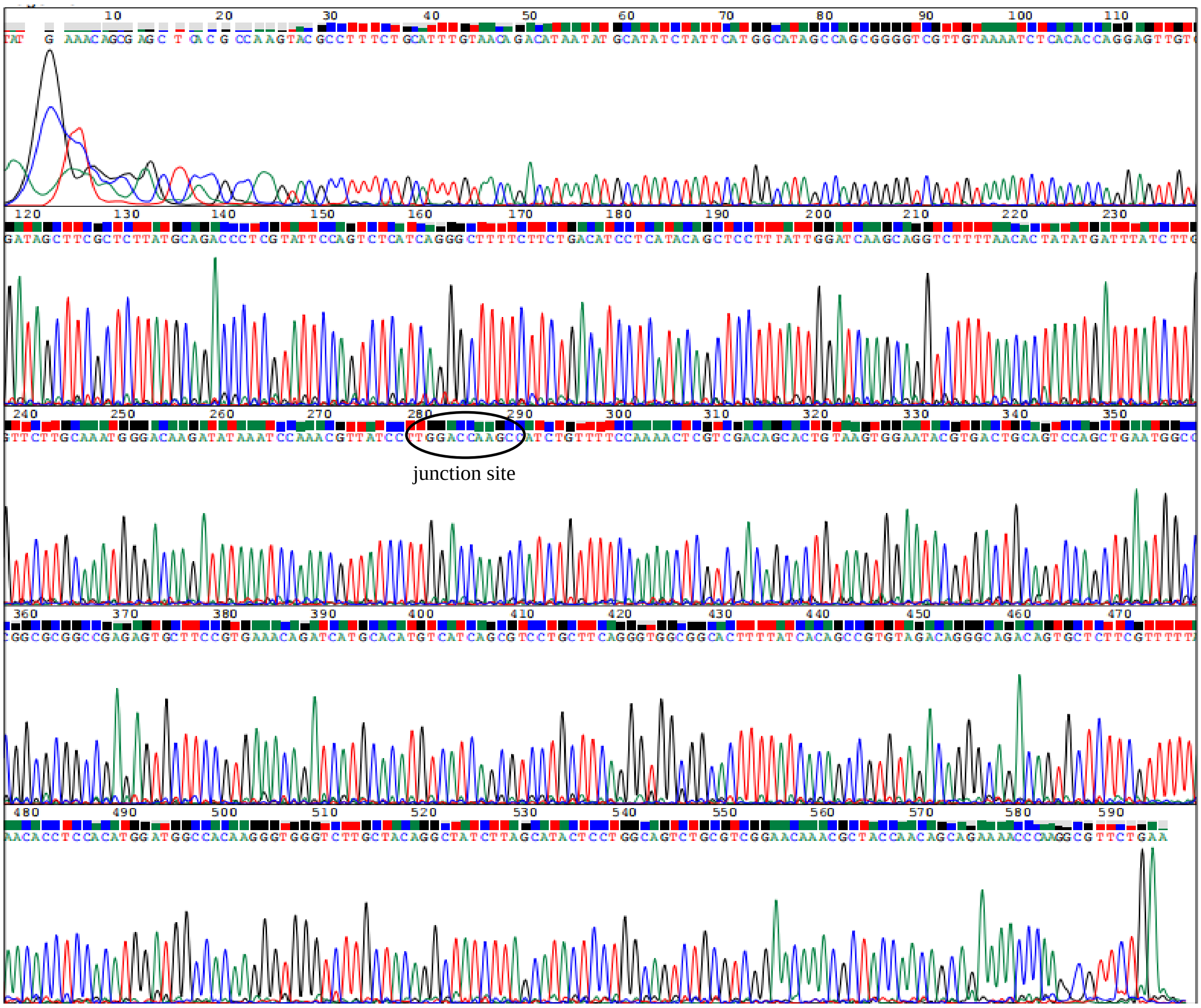

Sanger sequencing results of the RT-PCR product of oar_circ_0007733

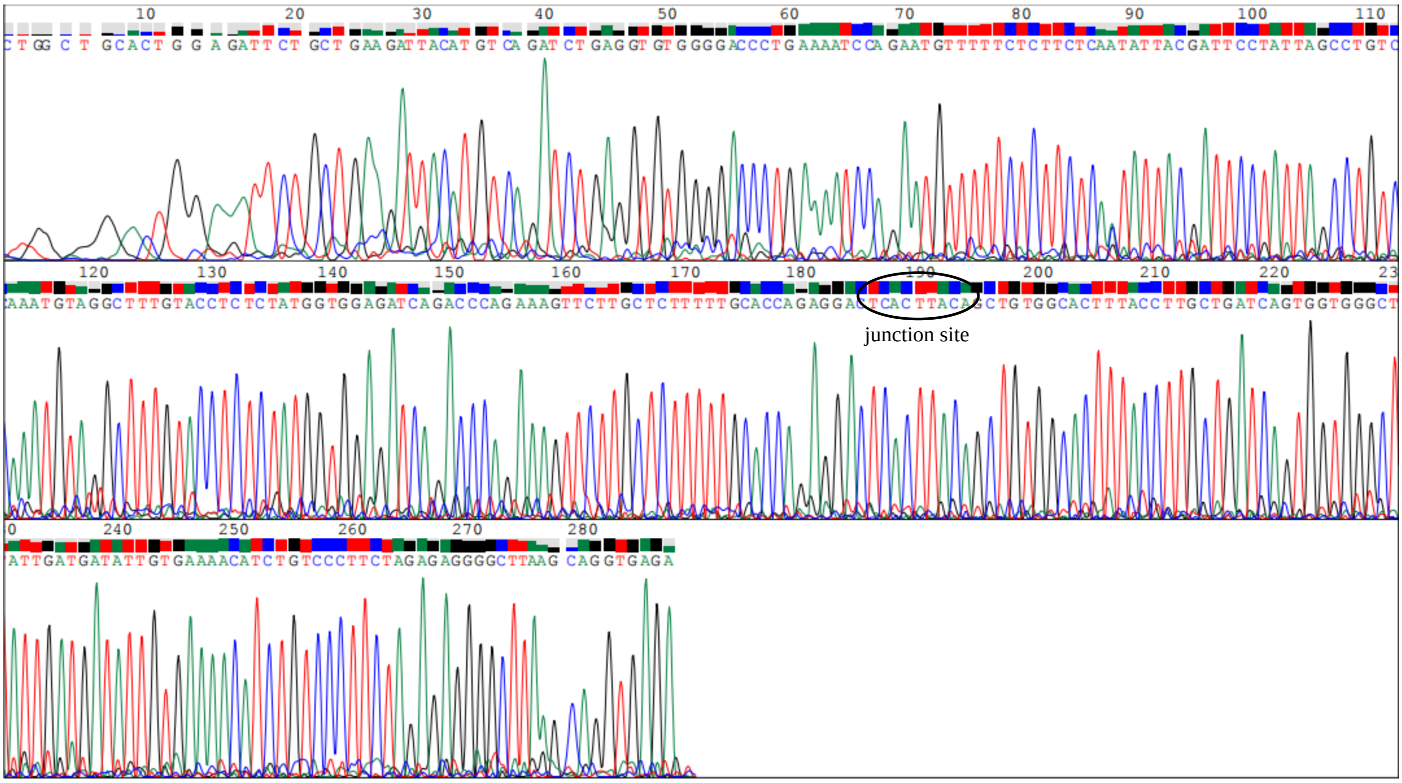

Sanger sequencing results of the RT-PCR product of oar_circ_0019258

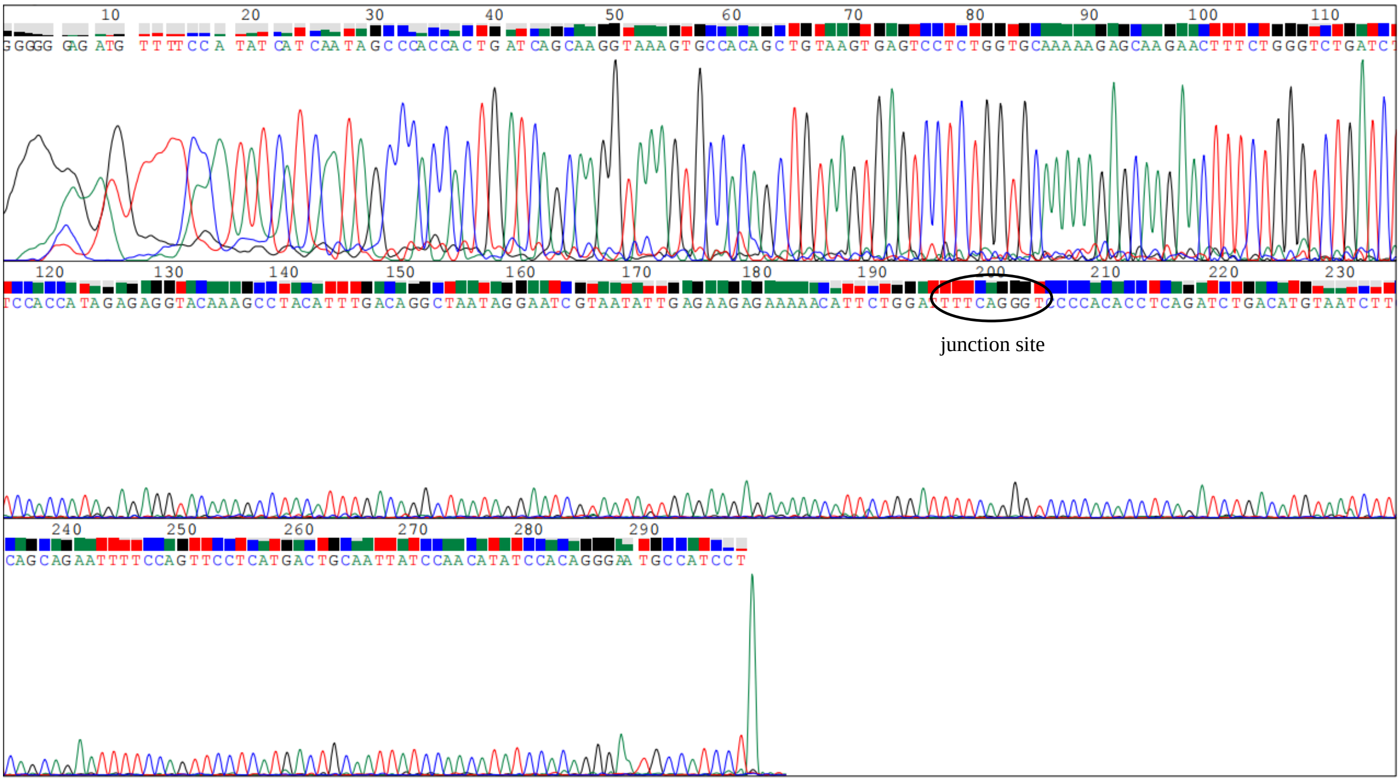

Sanger sequencing results of the RT-PCR product of oar_circ_0007731

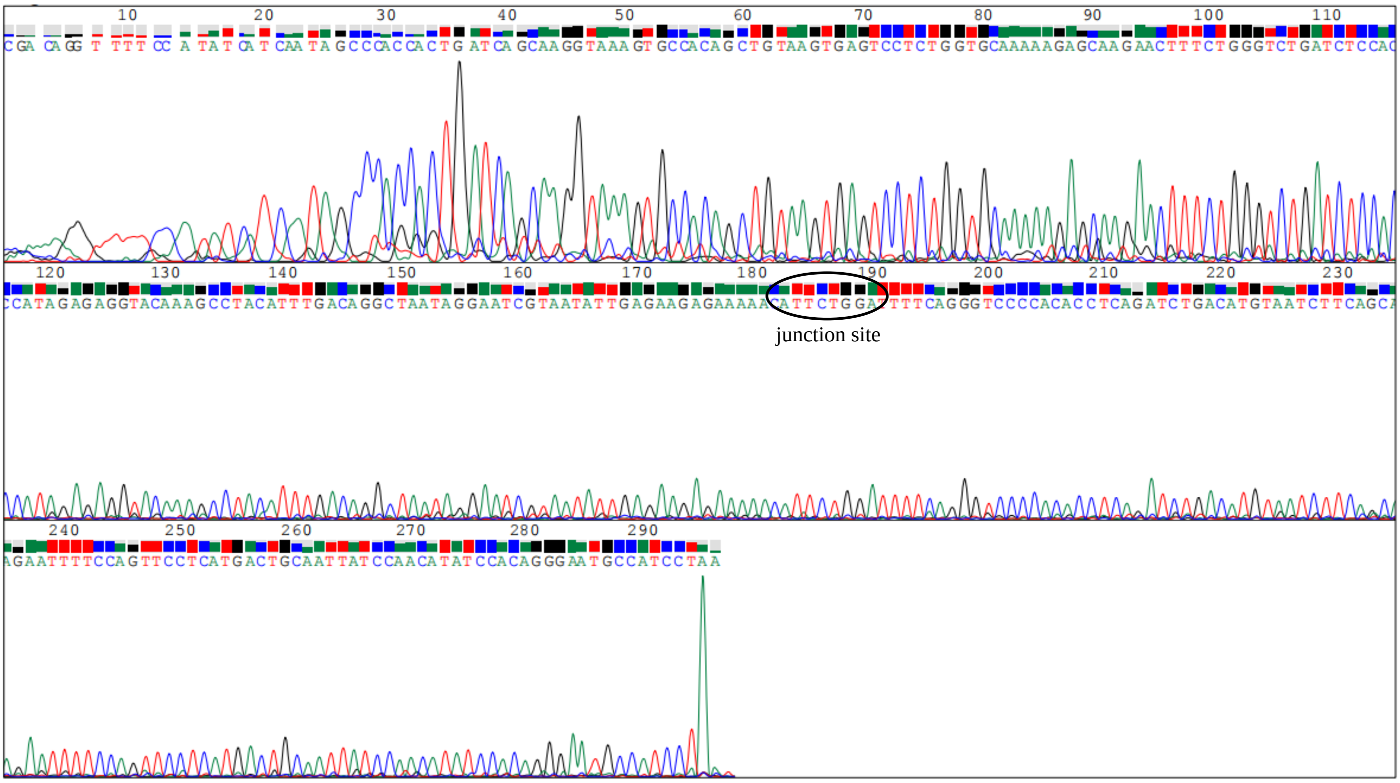

Supplement: Supplementary file 1 [file animals-14-00198-s001.zip › Figure S1 Sanger sequencing results of the RT-PCR products of circRNAs.pdf]
